# Distinct Age-Specific miRegulome Profiling of Isolated Small and Large Intestinal Epithelial Cells in Mice

**DOI:** 10.3390/ijms22073544

**Published:** 2021-03-29

**Authors:** Juneyoung Lee, Attayeb Mohsen, Anik Banerjee, Louise D. McCullough, Kenji Mizuguchi, Motomu Shimaoka, Hiroshi Kiyono, Eun Jeong Park

**Affiliations:** 1 Division of Mucosal Immunology, Department of Microbiology and Immunology, The Institute of Medical Science, The University of Tokyo, 4-6-1 Shirokanedai, Minato-ku, Tokyo 108-8639, Japan; juneyoung.lee@uth.tmc.edu (J.L.); kiyono@ims.u-tokyo.ac.jp (H.K.); 2 Department of Neurology, McGovern Medical School, The University of Texas Health Science Center at Houston, 6431 Fannin Street, Houston, TX 77030, USA; Anik.Banerjee@uth.tmc.edu (A.B.); Louise.D.McCullough@uth.tmc.edu (L.D.M.); 3 Laboratory of Bioinformatics, Artificial Intelligence Center for Health and Biomedical Research, National Institutes of Biomedical Innovation, Health and Nutrition, 7-6-8 Saito-Asagi, Ibaraki, Osaka 567-0085, Japan; attayeb@nibiohn.go.jp (A.M.); kenji@nibiohn.go.jp (K.M.); 4 Institute for Protein Research, Osaka University, 3-2 Yamadaoka, Suita-shi, Osaka 565-0871, Japan; 5 Department of Molecular Pathobiology and Cell Adhesion Biology, Mie University Graduate School of Medicine, 2-174 Edobashi, Tsu, Mie 514-8507, Japan; shimaoka@doc.medic.mie-u.ac.jp; 6 Department of Mucosal Immunology, The University of Tokyo Distinguished Professor Unit, 4-6-1 Shirokanedai, Minato-ku, The Institute of Medical Science, The University of Tokyo, Tokyo 108-8639, Japan; 7 Mucosal Immunology and Allergy Therapeutics, Institute for Global Prominent Research, Graduate School of Medicine, Chiba University, 1-8-1 Inohana, Chuo-ku, Chiba-shi, Chiba 260-8670, Japan; 8 CU-UCSD Center for Mucosal Immunology, Allergy and Vaccines (cMAV), Division of Gastroenterology, Department of Medicine, School of Medicine, University of California San Diego, 9500 Gilman Dr. MC 0063, San Diego, CA 92093-0063, USA

**Keywords:** intestinal epithelial cells, aging, microRNA, cellular senescence

## Abstract

The intestinal epithelium serves as a dynamic barrier to protect the host tissue from exposure to a myriad of inflammatory stimuli in the luminal environment. Intestinal epithelial cells (IECs) encompass differentiated and specialized cell types that are equipped with regulatory genes, which allow for sensing of the luminal environment. Potential inflammatory cues can instruct IECs to undergo a diverse set of phenotypic alterations. Aging is a primary risk factor for a variety of diseases; it is now well-documented that aging itself reduces the barrier function and turnover of the intestinal epithelium, resulting in pathogen translocation and immune priming with increased systemic inflammation. In this study, we aimed to provide an effective epigenetic and regulatory outlook that examines age-associated alterations in the intestines through the profiling of microRNAs (miRNAs) on isolated mouse IECs. Our microarray analysis revealed that with aging, there is dysregulation of distinct clusters of miRNAs that was present to a greater degree in small IECs (22 miRNAs) compared to large IECs (three miRNAs). Further, miRNA–mRNA interaction network and pathway analyses indicated that aging differentially regulates key pathways between small IECs (e.g., toll-like receptor-related cascades) and large IECs (e.g., cell cycle, Notch signaling and small ubiquitin-related modifier pathway). Taken together, current findings suggest novel gene regulation pathways by epithelial miRNAs in aging within the gastrointestinal tissues.

## 1. Introduction

Aging is a risk factor for many chronic diseases and contributes to systemic disruptions that increase the prevalence of illness [1]. Aging predisposes individuals to a heightened risk of both mortality and morbidity from a multitude of diseases which decrease quality of life in the elderly [2]. Though the precise definition of aging is still unclear, it has been established that with aging, there is a gradual accumulation of deleterious factors within host tissues, triggering various age-related cell death pathways leading to loss of function at the cellular levels [3,4,5]. Recent studies have shown that aging induces a low-grade chronic inflammation referred to as “inflammaging”, which is associated with poorer functional recovery following the onset of acute injuries in the elderly [6]. Such age-induced functional decline, especially within the gastrointestinal (GI) tract and its local immune system, directly contributes to higher rates of systemic inflammation and infection [7,8]. Therefore, understanding the mechanisms of tissue senescence regulated by epigenetic and cellular pathways is essential for the development of novel therapeutic strategies to treat age-related diseases.

In the perspective of GI physiology and morphology, several studies have shown significant alterations on both the tissue and cellular levels of inflammation associated with aging. Aging results in various phenotypic changes in the intestines, such as a compromised mucosal barrier, shifts in microbial diversity, and decreased beneficial microbial metabolites [9,10]. Moreover, aging impairs the functional epithelial layer in the gut and leads to increased permeability [10,11]. Moreover, it causes abnormal translocation of toxic luminal contents such as pathogenic bacteria into the host intestinal tissue, leading to both local and systemic immune activation [12,13]. Thus, the reduction of gut barrier function seen with aging often defined as “leaky gut” [14] is thought to be a prerequisite for the initiation of gut-primed inflammation in various diseases.

Intestinal epithelial cells (IECs) serve as a protective barrier between the luminal environment and host tissue [15]. IECs convey luminal signals into the resident immune cells of the lamina propria and maintain tissue homeostasis by mounting appropriate immune responses. The production of physical, biochemical and immunological components by strategically positioned specialized cell types is a salient feature of IECs that coordinates mucosal immunity in response to the dynamic milieu of the intestinal luminal environment [15,16]. IECs also express distinct classes of toll-like receptors (TLRs) within the crypt–villus axis that directly bind to microbial products and serve as luminal sensors that promote appropriate inflammatory cues for epithelial cell homeostasis [17]. Microbe-induced ligation of TLRs and several downstream pathways regulate epithelial turnover, production of cytokines and proliferation of progenitor and intestinal stem cells (ISCs) [18]. Dysregulation of these IEC-specific protective functions and signaling pathways increases susceptibility to intestinal inflammation and infection, especially in the aged population [18]. However, a comprehensive understanding of age-associated epigenetic regulators that alter IEC genes remains to be determined.

MicroRNAs (miRNAs), a class of small endogenous non-coding RNAs, are key players in controlling essential biological processes in various tissues including the intestines [19,20,21,22]. The regulatory roles played by miRNAs are progressed as follows: (1) binding to complementary sequences within the 3′-untranslational region (3′-UTR) of target genes, (2) downregulating messenger RNA (mRNA) expression of those key molecules in a posttranscriptional manner and (3) consequently affecting a variety of pathophysiological conditions [23,24,25]. In the intestines, those miRNAs have been recognized as the integral regulators or the targets to inflammatory disorders [20,26]. 

To understand the effect of aging progression on IEC genes and their potential regulators, we profiled and characterized miRNAs on isolated viable IECs from both small (small IECs) and large intestines (large IECs) of both young (two-month-old) and aging (12-month-old) mice. Next, in order to determine their biological relevance, we analyzed (1) age-specific IEC miRNAs, (2) region-specific IEC miRNAs (small IECs vs. large IECs) and (3) putative miRNA–mRNA networks and pathways by performing in silico analysis. Our data show that aging alone is sufficient to disrupt intestinal epithelial miRNAs in a regional manner. 

## 2. Results

### 2.1. Aging Predominantly Alters miRNA Transcriptome in Small IECs

IECs were isolated by cell sorting from the small (SI) and large intestinal (LI) tissues of two- and 12-month-old mice using a technique that we had previously established to isolate viable IECs with high purity (>98%) [20]. The expression profiles of miRNAs in the four distinct types of sorted IECs (i.e., small and large IECs from both young and aging mice) were determined using a predesigned targeted high-performance miRNA-based microarray [20]. Approximately 1900 miRNA signatures were detected based on their corresponding intensities. This stratification method led us to identify potential miRNA candidates that had differential expression in the progression of aging in a region-dependent manner.

**Figure 1:**
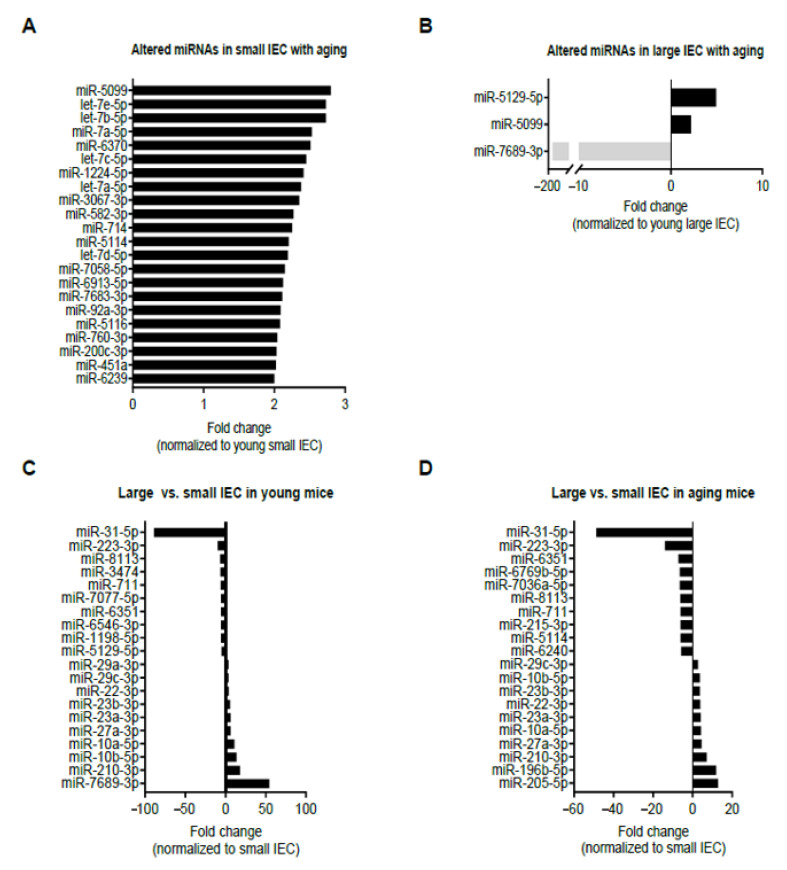
Bioinformatics analysis of differentially expressed miRNAs in isolated intestinal epithelial cells (IECs) from small (small IECs) (**A**) and large intestines (large IECs) (**B**) with aging, as well as large vs. small IECs in young (**C**) and in aging (**D**) mice. Young (two-month-old) and aging (12-month-old) mice were used (*n* = 6 per group) and the samples were pooled prior to monitoring for 1900 miRNAs candidates using high-throughput microarray profiling. Positive fold change (FC of 2 or greater) indicates an upregulation with aging, while negative FC denotes a downregulation in the progression of aging. (**C**,**D**) Top 10 miRNAs with positive fold change (FC ≥ 2) indicate those which were upregulated in large IECs as compared with small IECs, while top 10 miRNAs with negative FC (≥ 2) denote those which were downregulated in large IECs as compared with small IECs in young (**C**) and aging mice (**D**).

In the discovery phase, the miRNA expression of small-IECs among young and aging mice was comparatively analyzed utilizing the criteria (see Methods for details) and a threshold cutoff of fold change (FC) of 2 or greater [20,27]. Our data showed 22 miRNA candidates, the majority of which belonged to the let-7 family of miRNAs (i.e., let-7a-5p, let-7b-5p, let-7c-5p, let-7d-5p and let-7e-5p), were upregulated in small IECs isolated from aging mice as compared to young small IECs (Figure 1A). Interestingly, none of the differentially expressed miRNAs were found to be downregulated with aging in small IECs. In contrast, in large IECs, we identified three miRNAs were differentially expressed with aging using the same selection criteria (FC of 2 or greater). In particular, aging elevated the expression levels of two miRNAs, miR-5129-5p and miR-5099, in large IECs, whereas an approximate 161-fold decrease in the expression of miR-7689-3p (940.7 vs. 5.8) was observed with aging (Figure 1B). Most notably, miR-7689-3p was the only identified miRNA decreased in IECs with aging. 

**Figure 2:**
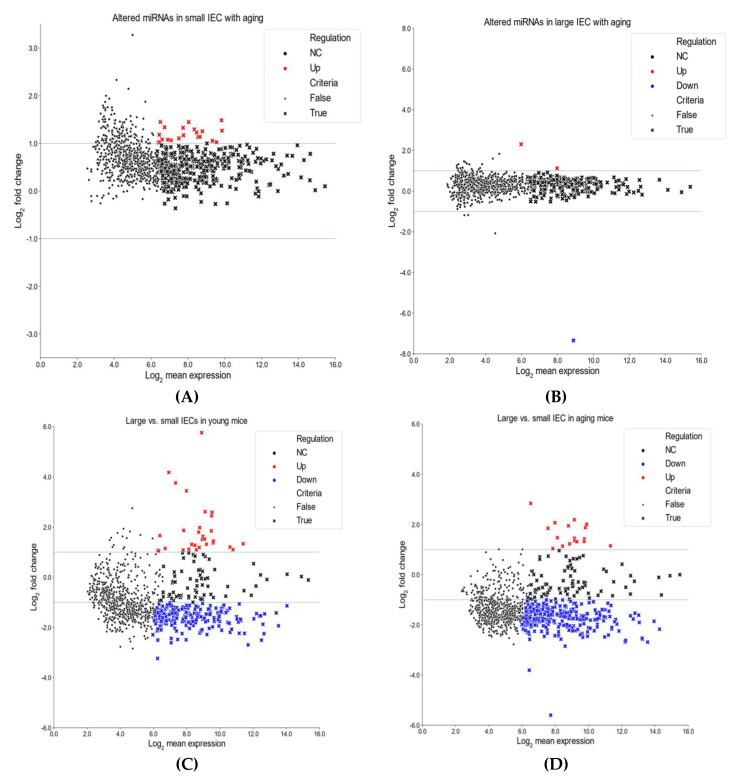
Scatter plots for miRNA log_2_ mean expression and log_2_ fold change (FC). Comparisons are shown for aging small IECs vs. young small IECs (**A**), aging large IECs vs. young large IECs (**B**), young large IECs vs. young small IECs (**C**) and aging large IECs vs. aging small IECs (**D**). Log_2_ FC is on the Y-axis, while log_2_ mean expression is on the X-axis. Those miRNAs that fulfill the criteria are represented by (x)s (see Methods for details). Red markers represent upregulated differentially expressed miRNAs (log_2_ FC > 1) and blue markers indicate downregulated differentially expressed miRNAs (log_2_ FC < −1).

We next compared miRNA profiles among small and large IECs to suggest unique region-based epigenetic modifications. Figure 1C,D shows the top 10 miRNAs that are either up/downregulated in large IECs compared with small IECs in young and aging mice. Specifically, several miRNAs, including miR-3474, miR-7077-5p, miR-6546-3p, miR-1198-5p and miR-5129-5p, were downregulated in large IECs of young mice. Further, in young mice miR-29a-3p and miR-7689-3p were upregulated primarily in large IECs. In aging mice, miRNAs, such as miR-6769b-5p, miR-7036a-5p, miR-215-3p, miR-5144 and miR-6240, were decreased, whereas miR-196b-5p and miR-205-5p were increased in large IECs as compared with small IECs. To provide further information on these assessments, we showed scatter plots for miRNA log_2_ mean expression and log_2_ FC (Figure 2). In this assay, four comparisons were performed in aging vs. young small IECs (A), aging vs. young large IECs (B), young large- vs. small IEC (C) and in aging large vs. small IECs (D). Those miRNAs that correspond to the aforementioned criteria are shown as x. Thus, these results demonstrate distinctive profiles of aging-associated miRNAs altered in gut epithelia.

### 2.2. Aging Confers More miRNA–mRNA Interactional Regulation Networks on Small IECs

Next, we sought to investigate the functional role of the identified candidate miRNAs to further elucidate their biological and physiological importance in the intestinal epithelium. As numerous reports have shown, miRNAs have a potential role in regulating multiple gene targets in the progression of inflammatory events in the gut [21,28,29]. We constructed interactional networks between the identified highest differentially expressed miRNA candidates (22 miRNAs in small IECs and three miRNAs in large IECs). Major continents and minor islands among the pool of networks were identified by centrality of degree and betweenness over the peripheral regions of interaction. Filtered miRNAs in the major continent were further analyzed to profile their putative target mRNAs using an analytics platform, miRNet 2.0. 

**Figure 3:**
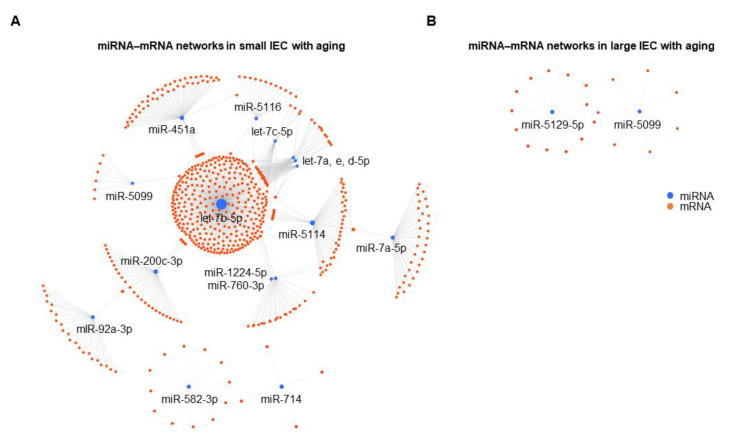
Putative miRNA–mRNA interaction networks in small (**A**) and large IECs (**B**) with aging. Differentially expressed miRNAs and their predicted mRNA transcript targets are illustrated as networks in both intestinal tissue types as generated by an analytics platform, miRNet 2.0. Each individual node represents a miRNA (blue) and an mRNA (orange), and gray lines signify a putative interaction.

As shown in Figure 3A, one continent and two island networks were identified in small IECs. The largest continent network incorporated 14 miRNAs (let-7b-5p, miR-451a, miR-5114, miR-7a-5p, miR-200c-3p, miR-92a-3p, let-7a-5p, let-7c-5p, let-7e-5p, let-7d-5p, miR-5116, miR-760-3p, miR-1224-5p and miR-5099; ordered by the degree or number of gene targets), followed by two distinct island interactions that were regulated by miR-582-3p (12 targets) or miR-714 (four targets). Interestingly, in the continent interactions, let-7b-5p had the highest number of predicted target mRNAs (352 targets), indicating its classification as a pivotal node. In contrast to small IECs, we observed two putative island networks in large IECs regulated by miR-5129-5p (13 targets) or miR-5099 (seven targets) (Figure 3B). Although expression of miR-7689-3p in large IECs was decreased with aging as shown in the microarray analysis (Figure 1B), the relevant networks related to this miRNA were not shown due to little information. To further analyze which genes are affected by the miRNAs in IECs, we investigated miRNet 2.0 and showed a much larger number of the mRNAs targeted by small IECs as compared to large IECs (Supplementary Tables S1 and S2, respectively). These results imply small IECs are more prone to be involved in epigenetic dysregulation with aging when compared with large IECs.

### 2.3. In Silico Analysis Identifies Distinct Pathways between Small and Large IECs That Are Associated with Aging

**Figure 4:**
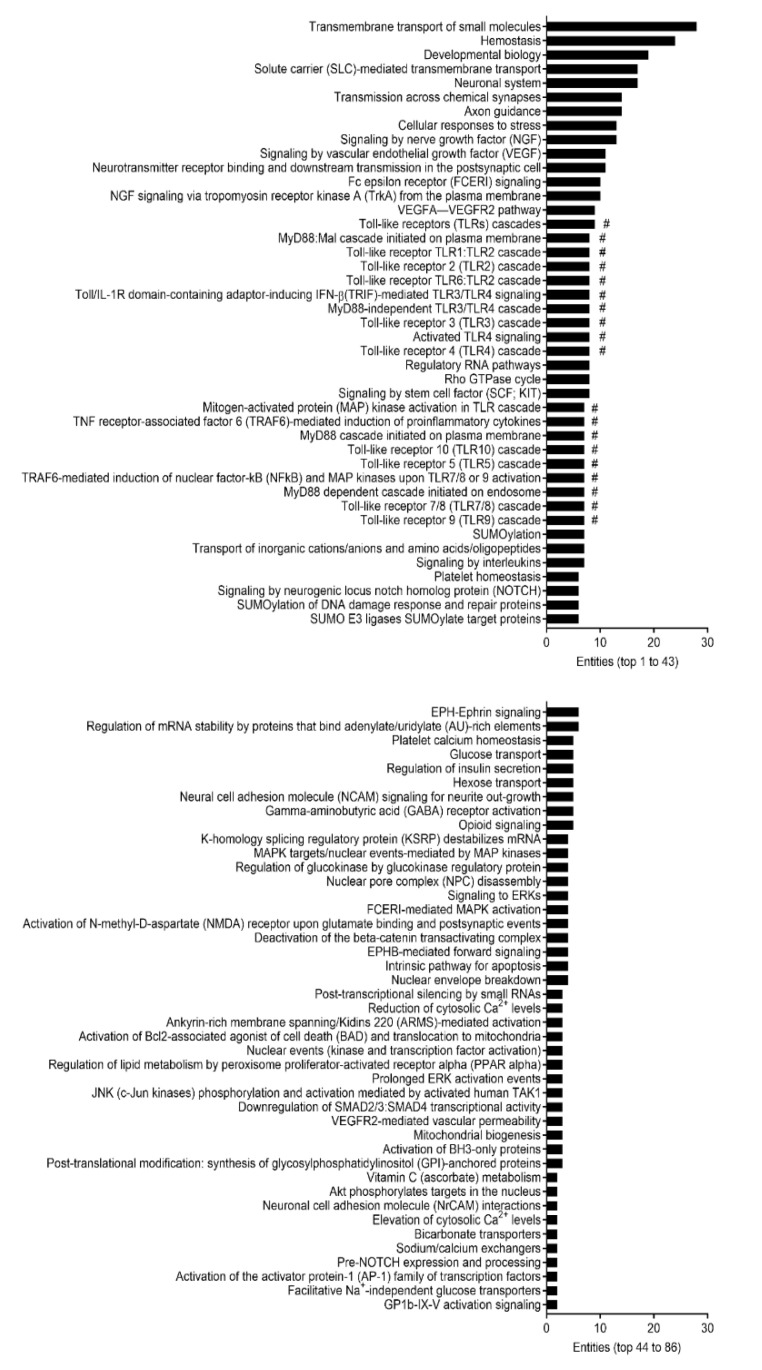
Pathway analysis of the predicted mRNA gene transcripts targeted by differentially expressed miRNAs in small IECs with aging. All the 86 identified pathways in small IECs regulated by 14 miRNAs filtered from interaction analysis were analyzed by Reactome and are shown based on the number of entities (i.e., the number of mRNA transcripts) involved. The # marks indicate toll-like receptor (TLR)-related pathways.

Numerous studies have demonstrated the effects of aging on gut morphology and physiology within intestinal tissues [8,10,12]. Therefore, we next examined underlying biomolecular pathways using Reactome, a multiple high-performance bioinformatics tool [30], which was provided as a functional analysis feature within miRNet 2.0 [31] to potentially pinpoint mechanisms that regulate cellular senescence in the intestinal epithelium. The Reactome analysis revealed that 86 putative pathways were regulated by the 14 filtered miRNAs following interaction analysis in small IECs based on the significance level (adjusted *p* < 0.05) (Figure 4). Intriguingly, 19 out of the 86 pathways identified were considered as being involved in TLR-related cascades in small IECs (Figure 4). 

**Figure 5:**
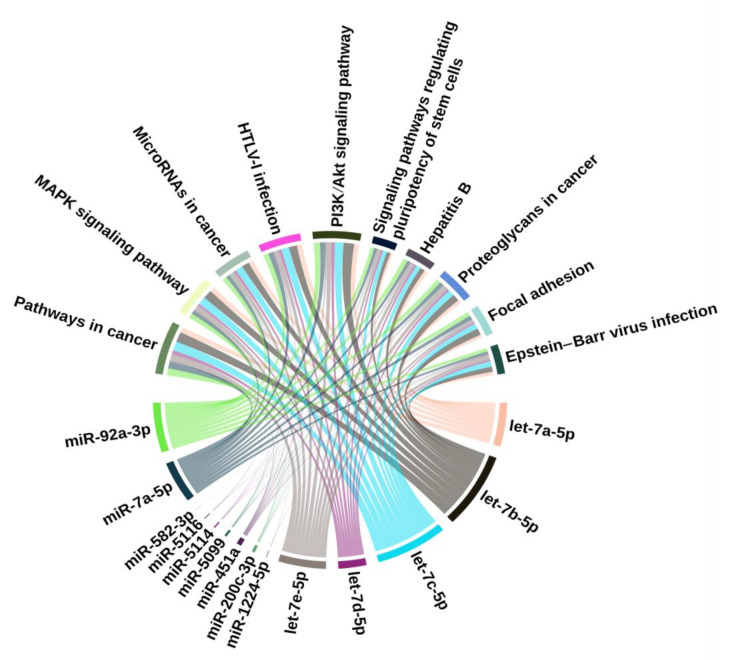
KEGG pathways enriched by the predicted mRNA gene transcripts targeted by differentially expressed miRNAs in small IECs with aging. The top 10 pathways in small IECs (top part) were regulated by 14 differentially expressed miRNAs (bottom part). The relation between the miRNAs and pathways is mapped through targeted mRNAs (not shown in the Figure because of their large number). The thickness of the connections represents the number of target mRNAs involved in the corresponding pathway.

We further performed systematic enrichment analysis using the Kyoto Encyclopedia of Genes and Genomes (KEGG) platform through the downstream pathway identifier in miRNet 2.0. The KEGG analysis revealed a total of 77 potential pathways that were regulated by the 14 filtered miRNAs within small IECs following interaction analysis, with a significance level of adjusted *p* < 0.05. In small IECs, the top 10 enriched pathways among the continent interactions were pathways in cancer, mitogen-activated protein kinase (MAPK) signaling pathway, miRNAs in cancer, human T-cell leukemia virus type 1 (HTLV-I) infection, PI3K/Akt signaling pathway, signaling pathways regulating pluripotency of stem cells, hepatitis B, proteoglycans in cancer, focal adhesion and Epstein–Barr virus infection (based on the number of hits) (Table 1). The KEGG analysis detected no pathways regulated by the two remaining island networks in small IECs. Figure 5 indicates the pathways enriched by the predicted gene transcripts targeted by the miRNAs differentially expressed in small IECs with aging. The top 10 pathways (upper part of the circle) were regulated by 14 differentially expressed miRNAs (lower part of the circle) in small IECs.

**Figure 6:**
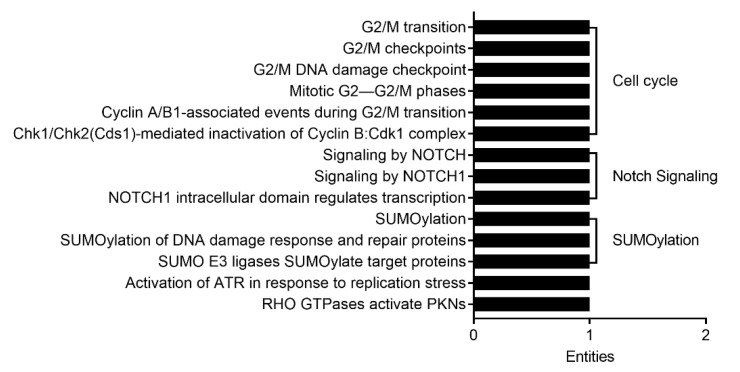
Pathway analysis of the predicted mRNA gene transcripts targeted by differentially expressed miRNAs in large IECs with aging. All of the 14 pathways regulated by miR-5129-5p in large IECs were analyzed by Reactome and were illustrated based on the number of entities (i.e., the number of mRNA transcripts) involved. Of note, miR-5129-5p was the only validated miRNA for the pathway analysis in large IECs. SUMO, small ubiquitin-related modifier; ATR, ataxia-telangiectasia-mutated and Rad3-related; and PKN, protein kinase N.

In large IECs, we found 14 pathways that were potentially regulated by miR-5129-5p (Figure 6) based on the same significance level (adjusted *p* < 0.05). It was possible to categorize miR-5129-5p-regulated events into three pathways (cell cycle, Notch signaling and SUMOylation). Due to no miR-5099-implicated pathways identified in the Reactome analysis, they were not shown. 

**Figure 7:**
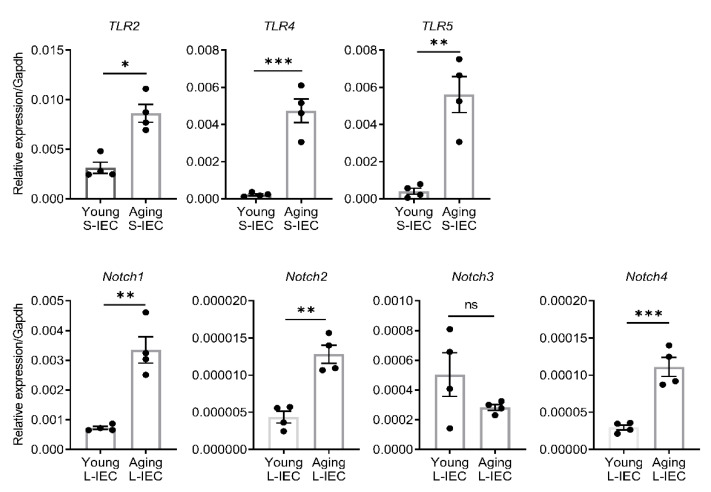
Validation of representative target gene expression in small and large IECs of young and aging mice. Small (S-IEC) and large IECs (L-IEC) were isolated from separate cohorts of young (two-month-old) and aging (12-month-old) mice through density gradient centrifugation using Percoll (see Methods for details). The expression levels of candidate genes proposed in the pathway analysis were examined by real-time quantitative PCR. Toll-like receptor (TLR) genes (*TLR2*, *TLR4* and *TLR5*) and Notch genes (*Notch1*, *Notch2*, *Notch3* and *Notch4*) were significantly upregulated in small (S-IEC) and large intestinal epithelial cells (L-IEC) of aging mice as compared with young mice. Gene for glyceraldehyde 3-phosphate dehydrogenase (Gapdh) was used as an endogenous control, in which the Student’s *t*-test was used. The data are presented as the means ± SEM. *n* = 4 per group. *, 0.01 < *p* < 0.05; **, 0.001 < *p* < 0.01; ***, *p* < 0.001; ns, not significant.

In the validation phase, we examined the expression levels of candidate genes such as TLRs in small IECs, which may potentially be activated by the identified miRNAs (Figure 4). Interestingly, a family of TLR genes including *TLR2* (2.8-fold), *TLR4* (21.0-fold) and *TLR5* (13.7-fold) were significantly upregulated in small IECs of aging mice as compared to those of young mice (upper panel of Figure 7). On the other hand, previous reports showed that Notch signaling can regulate self-renewal and differentiation of IECs [32,33,34]. Based on the results from Figure 6, we selected several Notch-related genes to validate their expression in large IECs. *Notch1* (4.6-fold), *Notch2* (3.0-fold) and *Notch4* (3.8-fold) were significantly increased with aging (lower panel of Figure 7). Together, these data suggest that aging may influence small and large IECs by altering unique classes of miRNAs and their corresponding gene targets. 

**Figure 8:**
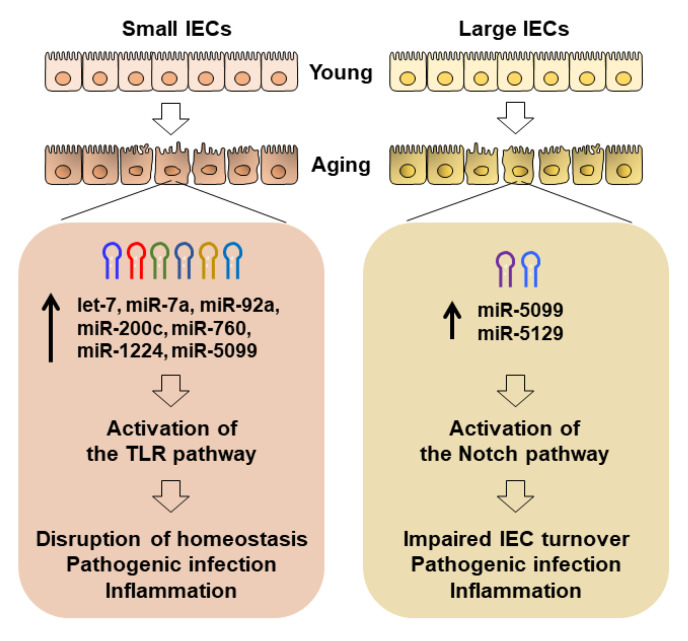
Proposed models for distinct miRNA-mediated aging effects on small and large IECs. A pool of multiple miRNAs including the let-7 family was increased in small IECs with aging. TLR pathways are potentially activated in small IECs, leading to the dysregulation of the host–bacteria balance, increased inflammation and infection. In large IECs, miR-5129 was elevated with aging. Genes that regulate IEC cycle and turnover (e.g., Notch genes) are possibly activated in large IECs with aging, leading to the disruption of IEC integrity, increased inflammation and infection.

Finally, a pool of multiple miRNAs including the let-7 family is expected to increase in small IECs with aging. TLR pathways are potentially activated in small IECs, leading to the dysregulation of the host–bacteria balance, increased inflammation and infection. On the other hand, miR-5129 was elevated in large IECs with aging. Some genes such as *Notch* that may regulate the IEC cycle and turnover are presumably activated in large IECs with aging, leading to the disruption of IEC integrity, increased inflammation and infection. Likewise, the proposed pathways illustrating distinct miRNA-engaged aging effects on facilitating pathogenesis in small and large IECs are shown in Figure 8.

## 3. Discussion

The intestinal epithelium plays a crucial role in mediating the crosstalk between the microbiome and the immune system within host tissue [15,16]. IECs are poised to directly interact with the microbiota and transduce signaling cascades to activate immune responses through a wide array of sensing molecules in response to environmental cues. Recent findings have demonstrated that aging of the host shifts the microbial composition (e.g., from youthful to detrimental), reduces epithelial integrity and enhances baseline inflammation in the intestinal tissues [9,10,12]; however, age-associated alterations in IECs at the cellular and epigenetic levels have not been well-defined and reports are extremely limited. To provide potential mechanisms for age-induced epithelial disruption, we utilized a high throughput-based microarray platform to identity differentially expressed miRNA candidates in aging across small and large IECs. Our data suggest that aging alters the profile of epithelial-specific miRNAs in the intestines in a region-dependent manner.

Aging elevated the expression of 22 miRNAs in small IECs following our selection criteria of FC of 2 or greater (Figure 1A). Interestingly, five miRNAs within the candidate pool (let-7a-5p, let-7b-5p, let-7c-5p, let-7d-5p and let-7e-5p) were a cluster of the let-7 family, which had previously been associated with cell differentiation and development [35]. In the intestines, the let-7 family of miRNAs are involved in regulating acute innate immune responses in response to potential pathogenic shifts in the microbiome. Matsushima et al. found that the let-7b expression is decreased in response to bacterial infection of the gastric mucosa, which induces acute and chronic inflammation [36]. Schulte et al. demonstrated that let-7b expression is downregulated in infected epithelial cells and immune cells with *Salmonella enterica*, an enteroinvasive pathogenic bacterium [37]. Furthermore, lipopolysaccharide-induced TLR4 signaling cascades were shown to repress the expression of the let-7 family and rescue cytokine production of IL-6 and IL-10, both of which are critical in bacterial infection [38]. However, this downregulation of let-7b did not coincide with the expression patterns seen in our age-related IEC-specific miRNA screen. The discrepancy of those expressions may possibly be due to the difference between “inflammaging” (low-grade chronic inflammation associated with aging) and the severe inflammatory conditions induced by an infection in the intestines. In line with our current findings that aging-associated let-7 expression is increased in IECs, Drummond et al. showed that the let-7 cluster was augmented in skeletal muscle biopsy of older subjects as compared to the younger ones [39]. Taken together, the levels of let-7 miRNAs in small IECs may be functionally associated with the maintenance of the host–microbial homeostasis in the intestines, suggesting that this normal state can be disrupted during aging through epigenetic regulatory roles played by those miRNAs. 

Moreover, miR-5099 was the only identified miRNA that was upregulated across both small and large IECs with the progression of aging (Figure 1A,B). Though our pathway analysis did not identify miR-5099-associated pathways, a distinct target prediction tool (TargetScanMouse 7.1) revealed that miR-5099 can potentially bind to the 3′-UTR of the Reg4 mRNA. Reg4, a member of the C-type lectin family, is an antimicrobial protein whose expression is limited to the intestinal epithelium [40]. Reg4 was expressed in deep crypt secretory cells of the colonic epithelium that regionally lacks Paneth cells, a specialized type of small IECs that serve to protect the stem cell niche by producing antimicrobial peptides [40]. Consequently, future investigations will need to figure out any aging-associated change in the level of Reg4 and its regulatory role in microbiota-associated inflammation. 

Distinct pathways were identified in S- and L-IECs in response to aging, suggesting diverse biological relevance of the identified miRNAs in a region-dependent manner. The Reactome analysis demonstrated that the pool of miRNAs in small IECs is involved in numerous physiologically relevant pathways; however, TLR-related cascades were predominant. As epithelial TLRs are crucial in regulating the clearance of pathogens, maintaining immune tolerance and promoting stemness/turnover in the host tissue to prevent inflammation and infection [18], our analysis indicated that aging itself may activate TLR pathways through miRNA alterations (Figure 4). Likewise, our validation study showed that aging significantly enhances the expressions of TLR genes in small IECs (Figure 6). Stimuli that may activate TLRs in a region-dependent manner within the context of aging is needed to be investigated in a future study.

The lifespan of IECs is tightly regulated and maintains intestinal homeostasis. Major signaling pathways such as Wnt/β-catenin, phosphatidylinositol-4,5-bisphosphate 3-kinase and Notch are crucial in IEC turnover [41,42,43]. Dysregulated IEC turnover has severe consequences, as epithelial barrier defects with excessive cell apoptosis enhance the entry of pathogens into host tissues [44]. Interestingly, the Reactome analysis showed that several pathways related to cellular turnover (cell cycles and Notch signaling) may be altered with aging in large IECs by targeting relevant genes (cyclins and Notch genes) (Figure 6). In consistence with our pathway analyses, Notch genes were significantly activated in large IECs of aging mice as compared to young mice (Figure 7). Notch signaling is essential for the maintenance of IEC differentiation [32,33,34], which implies that overactivation of Notch in aging may disrupt IEC turnover. Future studies will investigate the role of miRNAs in regulating Notch-related pathways that are involved in epithelial senescence.

Despite the analysis of miRNA profiles in small and large IECs of young and aging mice, it contains a limitation that mice of advanced age (over 24 months) were not utilized as a model for aging. Moreover, our analysis lacks the ability of estimating the *p*-value for significance measuring using statistical tests; however, our pooling of six samples per group reduced the effect of outliers and increased the power of the direct comparison [45]. Although the validation of the proposed target genes is needed, our data suggest that the identified miRNA candidates can target regulatory genes that are essential in maintaining epithelial homeostasis in the context of aging. In conclusion, this study provides an overview of IEC-specific miRNA networks that may regulate key genes and downstream pathways that potentially maintain the intestinal epithelium in aging. Our data provide a novel avenue for the development of miRNA-based interventions to restore intestinal homeostasis in aging.

## 4. Methods

### 4.1. Mice

Young (two-month-old) and aging (12-month-old) C57BL/6J mice were purchased from CLEA Japan (Tokyo, Japan). The mice were maintained in a specific pathogen-free animal facility (12:12-h light/dark cycle) at the Institute of Medical Science, the University of Tokyo (IMSUT). The mice were given water and food ad libitum. All the experiments performed were approved by the Institutional Animal Care and Use Committees of IMSUT, UTHSC and Mie University (27-6-2 approved on 11 June 2019).

### 4.2. IEC Isolation and Fluorescence-Activated Cell Sorting (FACS)

IECs were isolated as previously described [20]. Briefly, the SI and LI tissues were collected from mice after anesthesia and cervical dislocation. For the SI, Peyer’s patches were removed. The tissues were opened and rinsed thoroughly with ice-cold RPMI-1640 (Gibco, Grand Island, NY, USA) to remove luminal contents including feces. The rinsed tissues were cut into 1-cm-long slices and incubated in a digestion buffer (2 mM EDTA and 10% fetal calf serum in RPMI-1640) for 30 min at 37 °C. The resulting tissue suspension was filtered using a 70-μm cell strainer to remove tissue fragments. The filtered cell suspension was applied to a discontinuous gradient (25%/40%/75%) of Percoll (GE Healthcare Life Sciences, Chicago, IL, USA) and centrifuged at 780× *g* for 20 min at 22 °C. The interface between the 25% and 40% gradients were collected as “pre-sorting IECs.” The interface between the 40% and 75% gradients comprised mainly intraepithelial lymphocytes and was carefully removed to avoid any confounding effects of intraepithelial lymphocytes on miRNA expression of IECs. The “pre-sorting IECs” were stained with Via-Probe (BD Biosciences, San Jose, CA, USA), CD45 (30-F11, BioLegend, San Diego, CA, USA) and epithelial cell adhesion molecule (EpCAM) (G8.8, BioLegend). Via-Probe^-^CD45^-^EpCAM^+^ IECs were sorted using BD FACSAria III Cell Sorter (BD Biosciences) and used for miRNA microarray analysis.

### 4.3. RNA Isolation and Microarray Analysis for miRNAs

Total RNA was isolated from the sorted IECs using the Trizol reagent (ThermoFisher Scientific, Waltham, MA, USA) and immediately stored at −80 °C until the microarray analysis. The RNA isolated from the six samples per group was equally pooled in one pool before being labeled using a 3D-Gene^®^ miRNA Labeling Kit and hybridized on a 3D-Gene^®^ miRNA Oligo Chip (the number of mounted probes: 1900). After confirmation of the probe sequences, fluorescence in the chip was scanned using 3D-Gene^®^ Scanner (Toray, Kamakura, Japan). Probes with the minimum expression of less than 5 and the highest expression of less than 100 were excluded, and then fold changes were calculated correspondingly. The fold change of 2 was set as the threshold for differential expression criteria.

### 4.4. Construction of miRNA–mRNA Networks and Pathway Analysis

The miRNAs of the IECs obtained from the microarray analysis were applied to miRNet 2.0, a miRNA-centric network analytic platform [31] which incorporates miRBase (miRNAs) [46] and miRTarBase v8.0 (mRNA targets) [47], and were used to identify nodes and edges for the construction of the networks. The Reactome and KEGG analyses were obtained using the function explorer feature in miRNet 2.0 utilizing all the identified genes and the algorithm set to hypergeometric test. The data analysis was performed using the R statistical programming language and the plots were created using the seaborn package from Python and the circlize package from R [48].

### 4.5. Target Prediction

The predicted regulatory mRNA targets of the miRNAs were analyzed using TargetScanMouse 7.1, a public repository [19].

### 4.6. Real-Time Quantitative PCR (RT-qPCR)

Total RNA was isolated from IECs using Trizol (Invitrogen, Carlsbad, CA, USA). One microgram of RNA was used for cDNA synthesis using an iScript^TM^ Reverse Transcription Supermix for RT-qPCR (Bio-Rad, Hercules, CA, USA). RT-qPCR was performed using an SsoAdvanced Universal SYBR Green Supermix (Bio-Rad) in a CFX96 Touch Real-Time PCR Detection System (Bio-Rad). *Gapdh* was used to normalize the gene expression levels. Primer sequences (5′→3′) used in this analysis were as follows: *Gapdh*, TGTGTGCGTCGTGGATCTGA (forward) and TTGCTGTTGAAGTCGCAGGAG (reverse); *TLR2*, AAGAGGAAGCCCAAGAAAGC (forward) and CGATGGAATCGATGATGTTG (reverse); *TLR4*, ACCTGGCTGGTTTACACGTC (forward) and CTGCCAGAGACATTGCAGAA (reverse); *TLR5*, AAGTTCCGGGGAATCTGTTT (forward) and GCATAGCCTGAGCCTGTTTC (reverse); *Notch1*, CCGTGGCTCCATTGTCTACCT (forward) and CATCGGTGGCACTCTGGAA (reverse); *Notch2*, CCAAGCGGAAGCAAGCAT (forward) and GGCGCTTGTGATTGCTAGAGT (reverse); *Notch3*, TGCCAGAGTTCAGTGGTGG (forward) and CACAGGCAAATCGGCCATC (reverse); *Notch4*, GGTTTGCCAGCTCCTATTGG (forward) and CAGCCAGCATCAAAGGTGTAGT (reverse).

### 4.7. Statistical Analysis

The data are shown as the means ± standard error of the mean (SEM). The data were acquired using the two-tailed Student’s *t*-test for comparison of two groups. The *p*-values < 0.05 were considered significant. Statistical analysis was conducted with Prism 8 (GraphPad, San Diego, CA, USA). 

## Figures and Tables

**Table 1:** Top 10 KEGG pathways in small IECs with aging.

KEGG Pathway	Hits
Pathways in cancer	22
MAPK signaling pathway	20
MicroRNAs in cancer	18
HTLV-I infection	18
PI3K/Akt signaling pathway	18
Signaling pathways regulating pluripotency of stem cells	17
Hepatitis B	16
Proteoglycans in cancer	16
Focal adhesion	16
Epstein-Barr virus infection	16

## Data Availability

Not applicable.

## Supplementary Materials

The following materials are available online at https://www.mdpi.com/article/10.3390/ijms22073544/s1, Table S1. The potential targets of deferentially expressed miRNAs in small IECs with age; Table S2. The potential targets of deferentially expressed miRNAs in large IECs with age.
